# Triple atrial sensing during cardiac resynchronization

**DOI:** 10.1002/joa3.12297

**Published:** 2020-01-08

**Authors:** S. Serge Barold, Andreas Kucher, Rainer Halfenberg

**Affiliations:** ^1^ Department of Medicine University of Rochester School of Medicine and Dentistry Rochester NY USA; ^2^ BIOTRONIK SE & Co. KG Berlin Germany; ^3^ Marienkrankenhaus Lϋnen Germany

**Keywords:** cardiac resynchronization, implantable cardioverter‐defibrillator, pacemaker, Twiddler syndrome, ventricular tachycardia

## Abstract

This report describes a patient who underwent cardiac resynchronization complicated by a Twiddler syndrome. This caused triple atrial sensing and an inappropriate shock.

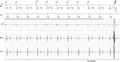

A 77‐year‐old man received a transvenous cardiac resynchronization device with cardioverter‐defibrillation capability (Lumax 640 HF‐T; BIOTRONIK). Ten months later, he presented with a shock. Figure 1 shows the data retrieved by interrogating the device. What is the interpretation of the recording and the mechanism of the shock? Why is atrial sensing present in all three channels?

**Figure 1:**
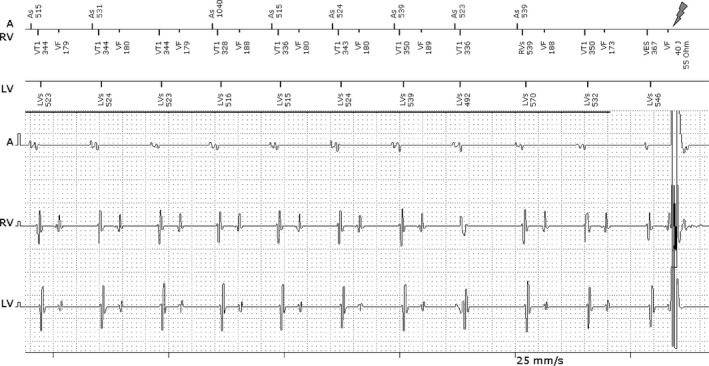
ICD shock resulting from sensing the near‐field atrial electrogram and the far‐field ventricular electrogram. See text for details

The device parameters and marker annotations were as follows: VT1 = programmed ventricular tachycardia interval for antitachycardia pacing (first therapy) 350 ms (170 bpm) and VF = ventricular fibrillation 270 ms (220 bpm). Therapy for VT1 was initiated with antitachycardia pacing (ATP); four bursts first followed by four ATP ramps and then a 40 J shock if ATP was unsuccessful.

In Figure 1, the marker channels are on top and the electrograms are below. A = atrium, RV = right ventricle, and LV = left ventricle. There is sinus rhythm at about 115 beats per minute and the third atrial beat is unsensed. There is also atrial undersensing before the shock on the right side of the tracing. Intermittent atrial undersensing is because of the atrial lead displacement. The right ventricular lead shows a double waveform consisting of the atrial and ventricular electrograms. On the right, there is a single episode of Mobitz type II AV block demonstrated by the loss of a ventricular electrogram. The right ventricular lead was displaced to the right atrium where the near‐field atrial electrograms and the far‐field ventricular electrograms are recorded. The right ventricular channel senses both the atrial and ventricular electrograms resulting in short ventricular intervals alternating with long ones. The short intervals (173‐188 ms), depicted by VF markers, correspond to the PR interval. The long intervals, depicted by VT1 markers, measure less than 350 ms (the VT1 interval) except for a longer interval after the non‐conducted atrial beat. The device delivers a shock at the end of the tracing as shown by the large baseline deflection. Note that all three leads demonstrate atrial sensing.

## 1 LEFT VENTRICULAR SENSING

Some implantable devices for cardiac resynchronization therapy (CRT) with cardioverter‐defibrillator capability, as the one in this report, can sense LV activity.^1^ LV sensing functionality was designed primarily to prevent pacing into the vulnerable period of the LV myocardium. Displacement of an LV lead toward the coronary sinus places it where left atrial activity may be sensed. There are no reports of far‐field atrial oversensing during sinus rhythm by a correctly positioned LV lead for CRT.^2^ Consequently, far‐field atrial sensing by an LV lead strongly suggests lead displacement toward the AV groove. Although far‐field atrial sensing did not involve the delivery of therapy it contributes to the description of triple atrial sensing.

## 2 TWIDDLER SYNDROME

**Figure 2:**
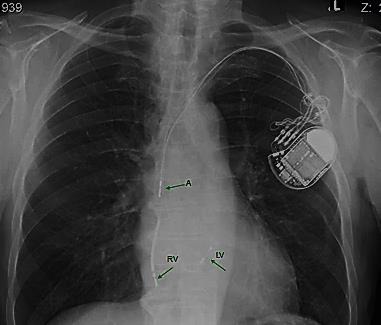
Chest X‐ray showing displacement of all three leads. The atrial lead (A) is in the superior vena cava and the right ventricular lead (RV) is in the right atrium. The left ventricular lead (LV) is displaced toward the coronary sinus but its location is not clearly discernible

Far‐field atrial oversensing in the right and left ventricular leads strongly suggests lead displacement from a Twiddler syndrome. Indeed, a chest X‐ray confirmed displacement of all three leads (Figure 2). The atrial lead was still in the right atrium. At surgery, all the leads were found to be grossly entangled, coiled, and with knots. In this respect, triple atrial sensing represents an important manifestation of Twiddler syndrome in patients with a cardiac resynchronization device capable of left ventricular sensing.

## 3 SHOCK DELIVERY

Inappropriate shocks in Twiddler syndrome often originate during sinus rhythm when a displaced right ventricular lead detects both the atrial electrogram and the far‐field ventricular electrogram. In a reported case, a displaced lead to the right atrium caused 49 inappropriate shocks.^3^ Less commonly, atrial fibrillation and a displaced lead in the right atrium are responsible for an inappropriate shock.^4^ Inappropriate shocks can precipitate serious arrhythmias in patients with preexisting ventricular tachyarrhythmias, while they are not always benign in patients without a history of cardiac arrhythmia. Excessive mechanical stress on the leads may cause insulation defects. These defects may be first recognized at the time of surgery. However, an insulation defect in the right ventricular lead may trigger a shock by producing noise that is interpreted as ventricular fibrillation by a device.^5^

## CONFLICT OF INTERESTS

The authors declare no conflict of interests for this article.

## References

[1] Barold SS , Kucher A . Understanding the timing cycles of a cardiac resynchronization device designed with left ventricular sensing. Pacing Clin Electrophysiol. 2014;37:1324–37. 25212575 10.1111/pace.12496

[2] Barold SS , Kucher A . Far‐field atrial sensing by the left ventricular channel of a biventricular device. Pacing Clin Electrophysiol. 2014;37:1624–9. 25139644 10.1111/pace.12482

[3] Spencker S , Poppelbaum A , Müller D . An unusual cause of oversensing leading to inappropriate ICD discharges. Int J Cardiol. 2008;129:e24–e26. 17669521 10.1016/j.ijcard.2007.06.088

[4] Garweg C , Alzand BS , Willems R . Twiddler syndrome causing an inappropriate implantable cardioverter‐defibrillator shock. Eur Heart J. 2014;35:516. 23918758 10.1093/eurheartj/eht275

[5] Nabeel Y , Gul S , Laiq Z . Twiddler syndrome: an unusual cause of repeated shocks by implanted cardioverter defibrillator. J Am Coll Cardiol. 2018; http://www.onlinejacc.org/content/71/11_Supplement/A2597 10.4103/HEARTVIEWS.HEARTVIEWS_45_19 PMC6791089 31620258

